# Supplementary material for: Myonuclear content regulates cell size with similar scaling properties in mice and humans
Source: Nat Commun. 2020 Dec 8;11:6288. doi: 10.1038/s41467-020-20057-8 (PMC7722898; doi:10.1038/s41467-020-20057-8)
Supplement: Supplementary file 1 — Reporting Summary [file 41467_2020_20057_MOESM1_ESM.pdf]

nature research

Corresponding author(s): Kristian Gundersen

Last updated by author(s): 16/09/2020

Reporting Summary

Nature Research wishes to improve the reproducibility of the work that we publish. This form provides structure for consistency and transparency in reporting. For further information on Nature Research policies, see Authors & Referees and the Editorial Policy Checklist .

Statistics

For all statistical analyses, confirm that the following items are present in the figure legend, table legend, main text, or Methods section.

n/a	Confirmed	
☐	☒	The exact sample size (*n*) for each experimental group/condition, given as a discrete number and unit of measurement
☐	☒	A statement on whether measurements were taken from distinct samples or whether the same sample was measured repeatedly
☐	☒	The statistical test(s) used AND whether they are one- or two-sided<br>*Only common tests should be described solely by name; describe more complex techniques in the Methods section.*
☐	☒	A description of all covariates tested
☒	☐	A description of any assumptions or corrections, such as tests of normality and adjustment for multiple comparisons
☐	☒	A full description of the statistical parameters including central tendency (e.g. means) or other basic estimates (e.g. regression coefficient) AND variation (e.g. standard deviation) or associated estimates of uncertainty (e.g. confidence intervals)
☐	☒	For null hypothesis testing, the test statistic (e.g. *F*, *t*, *r*) with confidence intervals, effect sizes, degrees of freedom and *P* value noted<br>*Give P values as exact values whenever suitable.*
☒	☐	For Bayesian analysis, information on the choice of priors and Markov chain Monte Carlo settings
☒	☐	For hierarchical and complex designs, identification of the appropriate level for tests and full reporting of outcomes
☒	☐	Estimates of effect sizes (e.g. Cohen's *d*, Pearson's *r*), indicating how they were calculated

Our web collection on statistics for biologists contains articles on many of the points above.

Software and code

Policy information about availability of computer code

Data collection	Olympus confocal microscope<br>Dragonfly spinning disc
Data analysis	Graphpad 8<br>Imaris software 8.2.1 (Bitplane)

For manuscripts utilizing custom algorithms or software that are central to the research but not yet described in published literature, software must be made available to editors/reviewers. We strongly encourage code deposition in a community repository (e.g. GitHub). See the Nature Research guidelines for submitting code & software for further information.

Data

Policy information about availability of data

All manuscripts must include a data availability statement. This statement should provide the following information, where applicable:

- Accession codes, unique identifiers, or web links for publicly available datasets
- A list of figures that have associated raw data
- A description of any restrictions on data availability

The data that support the findings of this study are available as Data source file

Field-specific reporting

Please select the one below that is the best fit for your research. If you are not sure, read the appropriate sections before making your selection.

Life sciences study design

All studies must disclose on these points even when the disclosure is negative.

Sample size	Sample size was determined by previous experience and according to guidelines of nested designs (see e.g. Aarts et al. 2014. A solution to dependency: Using multilevel analysis to accommodate nested data) to achieve at least an effective sample size of 40 single myofibers by assuming 10% variance between research objects.
Data exclusions	No data were excluded from the analyses
Replication	Experiments were performed on multiple cohorts of mice, with control mice in each cohort. All attempts of replication were successful.
Randomization	Mice and humans were randomly placed into experimental groups. For details regarding human participants see Psilander et al. 2019 J Appl Physiol 126: 1636-1645, 2019.
Blinding	Investigators were not blinded because the samples are easily distinguished due to genetic perturbation resulting in obvious reductions in myonuclear number

Reporting for specific materials, systems and methods

We require information from authors about some types of materials, experimental systems and methods used in many studies. Here, indicate whether each material, system or method listed is relevant to your study. If you are not sure if a list item applies to your research, read the appropriate section before selecting a response.

Materials & experimental systems

n/a	Involved in the study	
☒	☐	Antibodies
☒	☐	Eukaryotic cell lines
☒	☐	Palaeontology
☐	☒	Animals and other organisms
☐	☒	Human research participants
☒	☐	Clinical data

Methods

n/a	Involved in the study	
☒	☐	ChIP-seq
☒	☐	Flow cytometry
☒	☐	MRI-based neuroimaging

Animals and other organisms

Policy information about studies involving animals; ARRIVE guidelines recommended for reporting animal research

Laboratory animals	NMRI female mice (postnatal day 70-77). Myomaker loxP/loxP and Pax7-CreER mice are on the C57Bl6 strain. Both female and male were used from postnatal day 6 through 5 months of age.
Wild animals	No wild animals were used in this study.
Field-collected samples	No field-collected samples were used in this study.
Ethics oversight	All animal experiments were approved by the Norwegian Animal Welfare authorities according to the Norwegian Welfare Act. For developmental studies, all animals procedures were approved by Cincinnati Children's Hospital Medical Center's Institutional Animal Care and Committee.

Note that full information on the approval of the study protocol must also be provided in the manuscript.

Human research participants

Policy information about studies involving human research participants

Population characteristics	For analyses of biopsies of human subjects we re-used data from:<br>Psilander et al. 2019<br>J Appl Physiol 126: 1636-1645, 2019<br>First published April 11, 2019.
Recruitment	We included 7 male subject from the original study by Psilander et al. 2019
Ethics oversight	In the original study human participants were carefully informed about the experimental design, and possible risks related to the projects and gave written consent before entering the study. The study was approved by the Regional Ethics Committee of Stockholm, Sweden (DNR 2015/211-31/4), and was performed in accordance with Declaration of Helsinki.

Note that full information on the approval of the study protocol must also be provided in the manuscript.
